# New Common and Rare Variants Influencing Metabolic Syndrome and Its Individual Components in a Korean Population

**DOI:** 10.1038/s41598-018-23074-2

**Published:** 2018-04-09

**Authors:** Ho-Sun Lee, Yongkang Kim, Taesung Park

**Affiliations:** 1 Department of Statistics, Seoul National University, Seoul, 08826 Republic of Korea; 2 Daegu Institution, National Forensic Service, 33-14, Hogukro, Waegwon-eup, Chilgok-gun, Gyeomgsamgbuk-do Republic of Korea; 3 Interdisciplinary Program in Bioinformatics, Seoul National University, Seoul, 08826 Republic of Korea

## Abstract

To identify novel loci for susceptibility to MetS, we conducted genome-wide association and exome wide association studies consisting of a discovery stage cohort (KARE, 1946 cases and 6427 controls), and a replication stage cohort (HEXA, 430 cases and 3,264 controls). For finding genetic variants for MetS, with its components, we performed multivariate analysis for common and rare associations, using a standard logistic regression analysis for MetS. From the discovery and replication GWA studies, we confirmed 21 genome-wide signals significantly associated with MetS. Of these 21, four were previously unreported to associate with any MetS components: rs765547 near *LPL*; rs3782889 in *MYL2*; and rs11065756 and rs10849915 in *CCDC63*. Using exome chip variants, gene-based analysis of rare variants revealed three genes*, CETP*, *SH2B1*, and *ZFP2*, in the discovery stage, among which only *CETP* was confirmed in the replication stage. Finally, CETP D442G (rs2303790) associated, as a less common variant, with decreased risk of MetS. In conclusion, we discovered a total of five new MetS-associated loci, and their overlap with other disease-related components, suggest roles in the various etiologies of MetS, and its possible preventive strategies.

## Introduction

Metabolic syndrome (MetS) is defined as a combination of several clinical features, including central obesity, high blood pressure, elevated circulating levels of fasting glucose, high triglyceride (TG) levels, and low concentrations of HDL-cholesterol (HDLc). Since these features associate with increased risk of cardiovascular disease and type-2 diabetes, their increased worldwide prevalence and incidence are alarming. Moreover, ethnic disparities in the prevalence of MetS have been described^1^. Using the National Cholesterol Education Program’s MetS definition, its prevalence among U.S. adults was reported to be 23.8% in Caucasians, 21.6% in African Americans, and 31.9% in Mexican Americans^2^. Likewise, the prevalence of MetS in Korea has also been steadily increasing, from 24.9%, in 1998, to 31.3%, in 2007.This prevalence is relatively high compared to those of other Asian countries, such as Japan (16.5% in 2004), Taiwan (25.5% in 2008), and China (21.3% in 2009)^3,4^. Studies from Asian and European cohorts have also demonstrated high degrees of heritability in the individual components of MetS^5^; for example, monozygotic twin studies showed, heritability of MetS to be 50–60% in Korea^6^. Therefore, a significant role of genetic factors in the development of MetS, in Korean populations, can be compared to other ethnic and racial groups.

In one investigation of lipid metabolic traits, components of MetS, a genome-wide linkage analysis correlated the three lipid traits of HDLc, TGs, and low-density lipoprotein particle size, thus supporting the hypothesis of genetic pleiotropy as a source of correlation among metabolic components^7^. However, the associations were reported to differ between the same components, in the case of MetS^8^, as single nucleotide polymorphisms (SNPs) may associate with one MetS component and not with another. Therefore, genetic analyses are complicated by MetS being defined as an inherently heterogeneous pathology. This complexity has prompted the use of novel gene discovery methods such as factor analysis^8^, network-based enrichment analysis, or phenome-wide association study^9^. These have all been used for characterizing the genetic architecture of MetS, in addition to conventional logistic regression analysis (LR), with MetS as a binary response variable. Generally, dichotomization of several components into binary components, in these analyses, may result in loss of statistical power for finding genetic variants associated with MetS^10^. In addition, there are still several genetic variants that may cause even meta-analysis to have limited power, due to their small (but significant) effects on phenotype.

Although several recent genome-wide association (GWA) studies have identified common variants influencing combinations of MetS components^10–13^, it remains unclear whether observed genetic variants accurately reflect pleiotropic effects of genes in MetS, which may shed light on dysregulated mechanisms of the disease. Similarly, rare variants have gathered increasing attention as a possible alternative source of missing heritability for type-2 diabetes, and other metabolic diseases. Most MetS loci previously identified in population-based studies are common noncoding variants with small effects, and thus, analysis of rare or less common variants, are expected to find important determinants of MetS risk. To that end various exome chips were designed to identify functional variants that contribute to human traits, by focusing on less common (minor allele frequency (MAF) = 0.01–0.05), and rare (MAF < 0.01) variants that alter amino acid sequence, which were not included in previous genotyping arrays. Therefore, identification of rare coding variants may result in information explaining loss of function, and may provide causative variants of MetS.

To improve finding novel key genetic variants for MetS, we performed GWA and exome-wide association (EWA) studies of MetS in Korean populations, who were genotyped with SNP- and exome-chips simultaneously. Compared with standard-content GWA study arrays, the exome array has significantly increased marker density across the entire coding human exome, thus increasing power to detect disease associations located within the coding frame. In this study, we identified common and rare variants from a discovery stage for susceptibility of MetS, which was then replicated using an independent cohort, with significant genes identified by the MetS training set. In addition, our meta-analysis combined the discovery and replication studies for identifying new risk loci for MetS, using: (1) LR modeling for MetS as a binary outcome; and (2) multivariate analysis (MulA) of the components of MetS to reduce false positive results, and find unknown genetic variants for MetS.

## Results

### Common variants association study

#### Discovery stage

**Figure 1:**
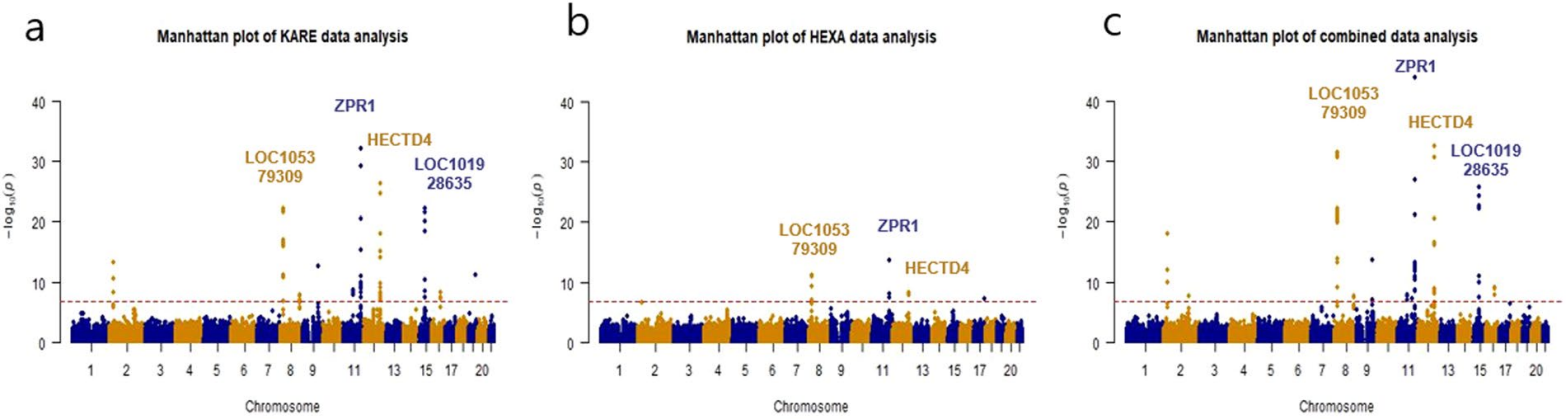
Manhattan plots showing the results of GWAS for MetS: (**a**) KARE, (**b**) HEXA and (**c**) Combined studies.

The quantile-quantile (QQ) plots of genome-wide P values, for multivariate analysis (MulA), deviated from the null distribution, due to strong associations observed for MetS in GWAS studies (Supplementary Figure S1). The prior quality control procedure showed no strong evidence of population stratification, in the KARE data^14^, and all samples with cryptic relatedness were removed before analysis^14^. The values of genomic inflation factors (ranging of 0.985–1.096 for KARE data, and 0.999–1.060 for HEXA data) did not show any evidence of population stratification, or other potential confounders. Figure 1 shows the Manhattan plots showing the results of MulA: (a) KARE, (b) HEXA and (C) Combined studies. The main hits are denoted by gene names. From the discovery GWAS, 77 loci were found significant (by Bonferonni correction, p < 1.6 × 10^−7^ represented by the red line in Fig. 1), among which 46 SNPs were also significant using LR (p < 0.05, Supplementary Table S2). Of the 44 newly identified common variants for MetS, 35 associated with at least one MetS component (from previous GWAS) (Table 1, Supplementary Table S1). The most significant association, in the discovery stage, was rs6589566, located within an intron of *ZPR1* (from MulA, P = 6.95 × 10^−33^), having a previously identified relationship with triglyceride (TG) levels, in Hispanic^15^ and European^16^ populations. More specifically, we identified this locus to associate with four components of MetS, namely FAG, HDLc, SBP, and TG (Table 2). Data from the discovery stage also confirmed the previously identified MetS-related variants rs12229654 (P = 9.05 × 10^−19^), in *LOC101929011*, rs11066280 (P = 5.91 × 10^−27^), in *HECTD4*, and rs780092 (P = 5.88 × 10^−14^), in *GCKR*, based on analysis by both LR and MulA (Supplementary Table S1). Interestingly, rs11066280 and rs2074356, in *HECTD4*, and rs12229654, near *LOC105369980*, associated with all MetS components, in the discovery stage. Of these, wild-type *HECTD4*, believed to encode an E3 ubiqutin ligase-4, has been reported to increase risk of type-2 diabetes [PMID: 26675016]. We also found 10 SNPs significantly associated (P < 0.05) with at least three components of MetS.

**Table 1:** Novel common SNPs^1)^ for metabolic syndrome achieving genome-wide significance in Korean population.

SNPs	Nearest Gene	Chr	CA/NCA	MAF	Position	Discovery			Replication			Meta-Analysis		
						P_MulA_	OR	P_OR_	P_MulA_	OR	P_OR_	P_MulA_	OR	P_OR_
rs11065756	CCDC63	12	A/G	0.17	109823177	9.58 × 10^−9^	0.88	<0.01	0.01	0.97	0.72	2.43 × 10^−9^	0.93	0.04
rs10849915	CCDC63	12	G/A	0.18	109818005	1.82 × 10^−8^	0.88	<0.01	0.02	0.96	0.69	7.86 × 10^−9^	0.92	0.03
rs17482310	LPL	8	T/C	0.21	19910554	9.55 × 10^−18^	0.87	<0.01	3.91 × 10^−7^	0.74	<0.01	6.68 × 10^−10^	0.80	2.68 × 10^−5^
rs3782889	MYL2	12	C/T	0.17	109835038	4.19 × 10^−9^	0.87	<0.01	0.01	0.96	0.64	1.48 × 10^−9^	0.91	0.02

^1)^SNPs not previously reported in GWAS of any other MetS traits. Chr, chromosome; MAF, minor allele frequency; OR, odds ratio; P_MulA_, P for multivariate analysis.

**Table 2:** Novel common SNPs^1)^ of metabolic syndrome for its six traits achieving genome-wide significance in Korean population.

SNPs	Locus	Chr	CA/NCA	Study	FAG		-HDL		TG		SBP		DBP		WC	
					beta	P	beta	P	beta	P	beta	P	beta	P	beta	P
rs11065756	CCDC63	12	A/G	Discovery	−0.07	1.06 × 10^−3^	0.07	3.86 × 10^−4^	−0.03	0.12	−0.06	3.66 × 10^−3^	−0.05	0.02	−0.07	3.51 × 10^−4^
				Replication	−0.04	0.19	0.11	5.38 × 10^−4^	0.05	0.13	−0.02	0.55	0.01	0.75	0.01	0.77
				Meta-Analysis	−0.05	1.92 × 10^−3^	0.09	3.4 × 10^−6^	0.01	0.08	−0.04	0.01	−0.02	0.08	−0.03	2.48 × 10^−3^
rs10849915	CCDC63	12	G/A	Discovery	−0.06	1.56 × 10^−3^	0.07	7.61 × 10^−4^	−0.03	0.13	−0.06	3.23 × 10^−3^	−0.04	0.03	−0.07	2.39 × 10^−4^
				Replication	−0.04	0.18	0.10	1.19 × 10^−3^	0.04	0.25	−0.02	0.43	0.002	0.95	0.002	0.94
				Meta-Analysis	−0.05	2.55 × 10^−3^	0.08	1.36 × 10^−5^	0.003	0.14	−0.04	0.01	−0.02	0.12	−0.04	2.11 × 10^−3^
rs765547	near LPL	8	T/C	Discovery	−0.02	0.23	−0.14	6.28 × 10^−14^	−0.12	2.19 × 10^−10^	−0.02	0.25	−0.01	0.48	0.025	0.19
				Replication	−0.02	0.56	−0.16	1.34 × 10^−8^	−0.13	8.89 × 10^−6^	−0.01	0.68	0.03	0.37	−0.04	0.15
				Meta-Analysis	−0.02	0.39	−0.15	4.17 × 10^−20^	−0.12	6.77 × 10^−14^	−0.004	0.47	0.007	0.50	−0.008	0.13
rs3782889	MYL2	12	C/T	Discovery	−0.07	4.6 × 10^−4^	0.07	4.73 × 10^−4^	−0.03	0.11	−0.06	3.25 × 10^−3^	−0.05	0.01	−0.08	1.95 × 10^−4^
				Replication	−0.04	0.23	0.11	6.15 × 10^−4^	0.05	0.14	−0.01	0.63	0.01	0.70	0.01	0.80
				Meta-Analysis	−0.05	1.09 × 10^−3^	0.09	4.67 × 10^−6^	0.01	0.08	−0.04	0.01	−0.02	0.06	−0.03	1.52 × 10^−3^

^1)^SNPs not previously reported in GWAS of MetS traits. Chr, chromosome; CA, coded allele; NCA, noncoded allele; P, P for multivariate analysis; FAG, fast glucose; TG, triglyceride; WC, waist circumference.

#### Replication stage and meta analysis

Here, we selected 46 SNPs from the discovery stage as candidates to carry forward to the replication stage, for further validation. Of the 46 loci, 21 were replicated with the same direction for MetS, except for rs6589567, near *APOA5*, rs6494005, in *LIPC*, and rs12708980, in *CETP* (all p < 0.05 with MulA and LR for each SNP, Supplementary Table S2). Of these 21,four were previously unreported to associate with any MetS components: rs765547 near *LPL* (fromMulA, P = 2.05 × 10^−22^; from LR, odds ratio (OR) = 0.80, P = 2.68 × 10^−5^); rs3782889 in *MYL2*(from MulA, P = 1.48 × 10^−9^; from LR, OR = 0.91, P = 0.02); and rs11065756 (from MulA, P = 2.43 × 10^−9^; from LR, OR = 0.93, P = 0.04) and rs10849915 (from MulA, P = 7.86 × 10^−9^; from LR, OR = 0.92, P = 0.03) in *CCDC63* (Table 1). Meta analysis, including GWA data, identified a total of six SNPs reaching genome-wide significance for any MetS component (Supplementary Table S3). Among these SNPs, five were newly identified variants for MetS, including rs16940212, rs495348, rs16940170 in *LOC101928635*, rs486394 in *LOC101929011*, and rs17482310, in *LOC105379309*. All of these SNPs were previously compiled in the NHGRI GWAS catalog for any MetS component (Supplementary Table S3). We also confirmed that rs486394 in *LOC105379309* demonstrated a significant (each P < 0.05) association with three or more MetS components.

### Rare variant association study

#### Discovery stage

We first considered the possibility that an aggregation of rare (or less common) coding alleles, in individual genes, contributes to variation in susceptibility of MetS risk, using EWAS data. To that end, we performed gene-based association analysis by SKAT. As a result, we identified an aggregation of rare coding alleles in *CETP* (p = 1.39 × 10^−32^), *SH2B1* (p = 1.47 × 10^−6^), and *ZFP2* (p = 3.08 × 10^−6^), that significantly associated with MetS, using Bonferonni correction (P = 4.11 × 10^−6^ (0.05/12153), Supplementary Table S4).

#### Replication stage and meta analysis

Here, we identified gene-based associations that were significant only for *CETP*, both in the replication stage (p = 1.33 × 10^−16^) and in meta-analysis (p = 2.05 × 10^−46^). However, neither *SH2B1* nor *ZFP2* significantly associated with MetS in the replication stage (Supplementary Table S4).

We next considered whether single variant testing of the genes identified by genome-based association study (GWAS)might influence MetS, such as rs5880, found in a previous GWAS for age-related macular degeneration, in Eastern Asians^17^. Therefore, we assessed each of the four protein-changing missense mutationsD459G, G331S, A390P, and E314K, for their effects on *CETP*, for MetS, By single variant association study, we successfully identified significant association of less common variants in *CETP*, with regard to MetS, namelyrs2303790 (MAF = 0.04, encoding D459G; from MulA, P = 1.73 × 10^−31^; from LR, OR = 0.71, p = 9.67 × 10^−4^), in the discovery stage (Table 3, Supplementary Table S5). In this locus, we found a strong association between D459G and increased HDLc levels (coefficient = −0.288), among MetS components. Replication evidence was compelling for CETP rs2303790 (from MulA, P = 3.56 × 10^−16^; from LR, OR = 0.71, p < 0.01).

**Table 3:** Association of the four observed CETP rare variants with MetS in the discovery stage.

Gene	Variants, Amino Acid Change	SNP	Location	CA/NCA	MAF	P_MulA_	OR	P_OR_
*CETP*	exon15, A1376G, D459G	rs2303790	57017292	G/A	0.0455	1.73 × 10^−31^	0.7131	9.67 × 10^−4^
	exon11, G991A, G331S	rs5881	57012012	A/G	0.0026	0.222	0.9781	0.9541
	exon12, G1168C, A390P	rs5880	57015091	C/G	0.0004	0.908	0.5016	0.5353
	exon10, G940A, E314K	rs140547417	57007387	A/G	7.47 × 10^−5^	0.987	1.70 × 10^−9^	0.9993

SNP, single nucleotide polymorphism; MAF, minor allele frequency; PMulA, P for multivariate analysis.

Our meta-analysis included EWA-identified single variants from the discovery and replication stages, and we confirmed that the rs2303790 less common variant showed genome-wide significance (from MulA, P* = *2.85 × 10^−17^; from LR, OR = 0.73, p = 0.001) for MetS (Supplementary Table S5).

## Discussion

Through discovery and replication, in various combinations with meta-analyses, we identified common variations of 36 new common loci, and one non-synonymous rare variant, i.e., rs2303790 in *CETP*, for MetS susceptibility in a Korean population. Of those 36, 29 were previously reported for at least one of MetS components, with the remaining 5 newly identified for MetS and its individual components.

Newly identified association signals, for MetS risk, within 8p21.3, was rs765547, located proximal to *LPL* (P_multi_ = 2.05 × 10^−22^; OR = 0.8, p = 2.68 × 10^−5^) in common variants. This variant also showed strong association, with HDLc and TG, in this study. This enzyme takes part in lipid metabolism, mediating hydrolysis of TG-rich lipoproteins, such as chylomicrons and VLDL. Persons deficient in LPL enzyme levels are characterized by severe hypertriglyceridemia, while those with elevated LPL showed lower TG levels^18^. Previously, LPL variantsrs1441756 and rs1297086 also associated with individual components of MetS^8^. Our result indicates that rs765547 has pleiotropic effects on TG and HDLc for MetS and bivariate effects of MetS. The mutant of this variant associated with increased HDLc, and decreased, TG levels. Although several studies shed light on LPL rs765547 association with MetS, other ethnic groups have remained elusive.

The coiled-coil domain within *CCDC63*, located in 12q24.11, included three significant SNPs (rs10849915, rs11065756, and rs2238149) in the discovery samples, and we found that two (rs10849915 and rs11065756) replicated with genome-wide significance in this study. Moreover, a previous study associated rs10849915 with intoxication behavior and alcohol consumption^19^. While the precise role of *CCDC63* in alcohol consumption remains unclear, alcohol is well known to cause hypertension, hypertriglyceridemia, and alcohol-related MetS components^20^. In addition, Go *et al*., reported that *CCDC63* rs11065756associated with type-2 diabetes^21^, as well as elevated glucose levels, two hours after an oral glucose challenge^22^. Our study also found that both rs11065756 and rs10849915 loci, within *CCDC63*, associated with the MetS components HDLc, TG, and waist circumference, as well as FAG.

We also found that *MYL2* variantrs 3782889 was involved in all MetS components, except for HDLc, in the discovery stage. Because *MYL2* encodes the regulatory myosin light chain that associates with cardiac myosin beta heavy chain, *MYL2* rs3782889 associated with risk of cardiovascular disease in a Korean population^23^. Similarly, we identified that the risk-specific allele, *MYL2* rs3782889, correlates with a decreased odds ratio (OR) of MetS (OR = 0.91, P = 0.02), suggesting that this locus might reduce MetS susceptibility, via increasing HDLc, and lowering TG levels. However, the functional role of the MYL2 protein, in the pathogenesis of MetS, has yet to be established.

One of our more the interesting findings was the identification of a less common mutation, polymorphism with in the *CETP* gene (D442G), associated with MetS. CETP contributes to lower HDLc since it transfers cholesteryl esters in HDLc to triglyceride-rich lipoproteins (such as VLDL and LDL) which can lead tohypertriglyceridemia^24^. A *CETP* variant, rs2303790, is an Asian-specific misssense variant defined as being polymorphic, and associating with elevated HDLc in Japanese and Chinese populations. The *CETP* allele (exon 15, nucleotide A > G, rs2303790, protein D442G) was reported to be a risk factor for age-related macular degeneration, with increased levels of HDLc, in East Asians^17^. Accordingly, we found that the D allele was significantly protective (OR = 0.73, p = 0.001), against MetS, because the D442G allelic mutation most strongly associated with increased HDLc. CETP D442G mutation was known to protect from coronary heart disease and Alzheimer’s disease^25^. Recently, protein-truncating variants of CETP reportedly reduced risk of coronary heart disease with higher level of HDLc^26^. However, the relationship between CETP D442Gmutation and coronary heart disease remains controversial.

Since MetS is defined by three or more altered components, different combinations of the individual components of MetS could have different effects on metabolic diseases. Some epidemiological studies suggested that specific clusters of MetS components may impact on higher risk of developing cardiovascular and metabolic disease^27^ and the occurrence of specific clustering of MetS differed across countries^28^. Similarly, it was reported that cluster of WC, TG, and HDL was mostly associated with the prevalence of MetS in Korea^29^ compared to WC and BP in China^30^, and TG and BP in Japan^31^. From Korean emigrant study^31^, the most influential components in diagnosing MetS in Korean was WC, TG and fasting glucose in men and WC, TG and BP in women living in Japan, and WC, TG and HDL for Korean living in China. Since various combinations of MetS components that share some basic pathophysiological patterns, genome-wide association studies for specific clustering of MetS components might improve interpreting etiology of MetS and the association between MetS and further progress of cardiovascular and metabolic diseases. Thus, further genetic studies on the clusters of MetS components might identify more relevant genes for understanding etiology of MetS. Despite strong evidence for genetic determinants of MetS and its components, however, only a few studies have been investigated for the association between genetic factor and clusters of MetS components. In our preliminary data, however, we found that the cluster analytical approach is not statistically feasible due to small sample sizes (data not shown). We will perform the cluster analysis of MetS components by integrating additional cohort data of Korean population in a near future.

For the KARE common variant data, we carried out SNP imputation with IMPUTE2 using the JPT/CHB component of HapMap as described in Cho, *et al*.^14^. However, the imputation method of rare variants has not well developed yet and has suffered from the low quality of imputation, as pointed out by Kim *et al*.^32,33^. Thus, we focused only on the genotyped rare and common variants.

In summary, our GWA and EWA studies of MetS provide new insights into the genetic mechanisms of MetS in Korean populations. This study greatly extends the number of known MetS trait loci, and moreover, demonstrates their significant relevance to MetS. The discovery of a total five new MetS-associated loci (four from our common-variant test, and one from our gene-based test and single variant test based on gene-based one) and their overlap with other disease-related etiologies of MetS and its risk, providing opportunities for preventive strategies against MetS. In addition, new variants, identified by our current study, have not previously been identified in large samples of MetS patients, or of other ethnic groups. Thus, although validation of the role of these variants in MetS in diverse ethnic groups is needed, these approaches are highly relevant to this worldwide scourge.

## Research Design and Methods

### Study Subjects

Our study samples consisted of 12,067 individuals from two independent cohort studies, which have both GWA and EWA datasets. For our GWAS analysis, the discovery stage cohort was comprised of 1946 cases, and 6427 controls, from the Korean Association REsource (KARE), part of the Korean Genome Analysis Project (KoGES), a large-scale GWA study, initiated in 2007, aiming to discover variants associated with numerous complex diseases and traits^34^. The replication stage cohort was derived from the Health EXAminee (HEXA), a part of the KoGES population-based cohort, initiated in 2001^34^. From HEXA, 3,694 samples, including 430 cases and 3,264 controls, were used for replication and validation of our model. We also performed EWA analysis, with our discovery stage, including 6,693 subjects, 1,530 cases and 5,163 controls, from KARE, while our replication stage included 3,429 subjects, 411 cases and 3,108 controls, from HEXA. All participants provided written informed consent and all study procedures were approved by the Institutional Review Board (IRB) of Seoul National University. The de-identified, individual-level genotype and phenotype data of KARE and HEXA samples were provided by the Korea Biobank Network, National Institute of Health, Korea.

### Metabolic syndrome (MetS)

MetS is defined in men and women, according to the U.S. American Heart Association/National Heart Lung and Blood Institute, and modified National Cholesterol Education Program Adult Treatment Panel III guidelines, requiring the presence of ≥3 of the following: (a) waist circumference ≥90 cm in men, or 80 cm in women; (b) triglyceride levels ≥150 mg/dL, or treatment for dyslipidemia; (c) HDLc levels <40 mg/dL in men or <50 mg/dL in women, or treatment for dyslipidemia; (d) systolic blood pressure (SBP) ≥130 mm Hg, or diastolic blood pressure (DBP) ≥85 mm Hg, or treatment for hypertension; or(e) fasting plasma glucose (FAG) levels ≥100 mg/dL, or treatment for diabetes mellitus. Practically, these guidelines differ from those of the National Cholesterol Education Program Adult Treatment Panel III, for the elevated fasting glucose criterion. MetS controls had ≤2 components. We excluded individuals having missing data for at least one MetS component.

### Genotyping and quality control for GWAS and EWAS

A total of 8,840 KARE study participants were genotyped using the Affymetrix Genome-wide SNP Array 5.0 (Affymetrix Inc., Santa Clara, CA, USA), which contains approximately 420,000 variants. The exclusion criterion for variants was as follows: Hardy Weinberg Equilibrium test P < 10^−6^, genotype call rates <95%, and monomorphic variants. For exome region analysis, 6,693 identical samples were genotyped using the Illumina HumanExome Chip v1.1 (Illumina, Inc., San Diego, CA, USA) exome chip. For quality control (QC), only monomorphic variants were excluded from rare variant analysis, using the same QC criteria performed for common variant analysis. From quality control data, we used 8,373 samples for Affymetrix SNP chip data, and 6,693 sample for Affymetrix exome chip data (Supplementary Table S6). Variants included in the analysis were 344,366 and 66,196 for the SNP- and exome-chips, respectively.

For identification of MetS-associated loci, a total of 3,696 HEXA study participants were genotyped using the Affymetrix Genome-wide SNP Array 6.0 (Affymetrix Inc., Santa Clara, CA, USA), which contains approximately 900,000 variants. We performed QC of the HEXA genotype dataset using the same procedure used for the KARE dataset. For exome chip analysis, a total of 3,429 samples were genotyped using the Illumina Human Exome Chip v1.1 (Illumina, Inc., San Diego, CA, USA). For QC, only monomorphic variants were excluded by the rare variant analysis. For quality control data, we used 3,694 samples from Affymetrix SNP chip data, and 3,429 samples from the Affymetrix exome chip data (Supplementary Table S6). Variants included in the analysis were 652,611and 49,921 for the SNP- and exome-chips, respectively.

### Statistical analyses

Prior to analysis, all possible MetS phenotypes were examined for departure from normal. In this study, all variables were log-transformed. Since HDLc negatively correlates with various MetS phenotypes, we used negative HDLc (-HDLc) in the analyses. The following methodologies were applied to identify SNPs or rare variants associated with single or multiple MetS phenotypes.

#### Single phenotype analysis

Single phenotype analysis with common variants:

1 $${\rm{logit}}({{\rm{p}}}_{{\rm{i}}})={\beta }_{0}+\sum _{k}{\gamma }_{k}{z}_{ik}+{\beta }_{j}{x}_{ij}$$

2 $${{\rm{y}}}_{{\rm{i}}}={\beta }_{0}+\sum _{k}{\gamma }_{k}{z}_{ik}+{\beta }_{j}{x}_{ij}+{{\epsilon }}_{i}$$

when analyzing a binary phenotype, MetS, we used logistic regression (LR), as defined in equation (1). The variable, p_i_, is the probability of a binary phenotype, *z*_*ik*_ is the *k*th covariate, and *x*_*ij*_ represents the *j*th genetic variant. In the same manner, to identify common variants associated with continuous phenotypes, such as components of MetS, linear regression was implemented as in equation (2). Logistic and linear regression models were fitted by plink to 1.0.7, by linear and logistic command, respectively.

Single phenotype analysis with rare variants: *Gene-based analysis for single phenotype*

3 $${\rm{logit}}({{\rm{p}}}_{{\rm{i}}})={\beta }_{0}+\sum _{k}{\gamma }_{k}{z}_{ik}+\sum _{j}{\beta }_{j}{x}_{ij}$$

4 $${{\rm{y}}}_{{\rm{i}}}={\beta }_{0}+\sum _{k}{\gamma }_{k}{z}_{ik}+{\beta }_{j}{x}_{ij}+\sum _{j}{\beta }_{j}{x}_{ij}+{{\epsilon }}_{i}$$

For rare variant analysis, for single phenotypes, we used the Sequence Kernel Association Test (SKAT)^35^. For the binary phenotype of MetS, LR was used as given in equation (3), and the linear regression was fit to equation (4), for each continuous component of MetS. Unlike common variant analysis, in both LR and regression models, *β*_*j*_ is the random effect of the *j*th variant of a gene. The significance of genetic effects was tested as to whether or not the variance component of *β*_*j*_ was zero. The SKAT model provides a gene-based p-value. To fit the model, we used the SKAT package with R version 3.3.0.

#### Multiple Phenotype Analysis

Multiple phenotype analysis with common nucleotide polymorphisms (SNPs): For multiple phenotype association with common variants, we adopted the scheme proposed by Oh *et al*.^36^. Multivariate regression was performed assuming that six MetS-related variables follow a multivariate Gaussian distribution. P-values were calculated from Wilk’s lambda statistics.

#### Gene-based analysis for multiple phenotypes with rare variant analysis

Rare variant analysis, for multiple phenotypes, was performed using Multivariate Association Analysis using Score Statistics (MAAUSS), as proposed by Lee *et al*.^37^. MAAUSS is a methodology, based on a variance component, which can be thought of as an extension of SKAT to multiple phenotypes. For this approach, two models are available: heterogeneous and homogeneous models. The former model allows different genetic effects on phenotypes, while the latter assumes common genetic effects on all phenotypes. To find genes significant for the multiple components of MetS, we performed a gene-based association test using MAAUSS. We likewise used less common and rare variants for each gene whose MAFs were <0.05 or <0.01. We considered P = 4.11 × 10^−6^ (0.05/12153) as the threshold of significance for the gene-based association analysis, according to the number of genes in our study.

## Electronic supplementary material

Supplementary Materials
