# Supplementary material for: New Common and Rare Variants Influencing Metabolic Syndrome and Its Individual Components in a Korean Population
Source: Sci Rep. 2018 Apr 9;8:5701. doi: 10.1038/s41598-018-23074-2 (PMC5890262; doi:10.1038/s41598-018-23074-2)
Supplement: Supplementary file 1 — Supplementary Materials [file 41598_2018_23074_MOESM1_ESM.docx]

**New Common and Rare Variants Influencing Metabolic Syndrome and Its Individual Components in a Korean Population**

Ho-Sun Lee^1,2^, Yongkang Kim^3^, and Taesung Park^1,3^

^1^Interdisciplinary Program in Bioinformatics, Seoul National University, Seoul 08826, Republic of Korea

^2^Daegu Institution, National Forensic Service, 33-14, Hogukro, Waegwon-eup, Chilgok-gun, Gyeomgsamgbuk-do, Reublic of Korea

^3^Department of Statistics, Seoul National University, Seoul 08826, Republic of Korea

Corresponding author: Taesung Park, Department of Statistics, Seoul National University, Seoul 08826, Republic of Korea; email: tspark@stat.snu.ac.kr; telephone:82-2-880-9168; fax:82-2-880-6693


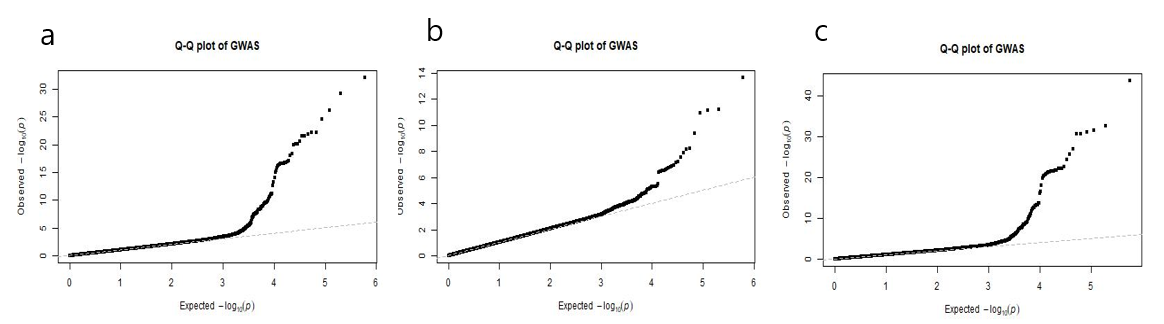


Supplementary Figure S1. Quantile-Quantile plot showing the results of GWAS for MetS (a) KARE, (b) HEXA, and (C) combined.

Supplementary Table S1. The newly identified common variants for metabolic syndrome in the discovery stage (MulA P<1.6 $\times$ 10^-7^, LR P<0.05)

| SNP | Traits | Nearest Gene | MAF | Position | MulA_p | OR | OR_P | **FAG** | | **-HDLc** | | **TG** | | **SBP** | | **DBP** | | **WC** | |
| --- | --- | --- | --- | --- | --- | --- | --- | --- | --- | --- | --- | --- | --- | --- | --- | --- | --- | --- | --- |
|  |  |  |  |  |  |  |  | beta | p | beta | p | beta | p | beta | p | beta | p | beta | p |
| **rs6589566** | TG | *ZPR1* | 0.22 | 116157633 | 7.0E-33 | 1.23 | 1.2E-06 | 0.049 | 0.009 | 0.135 | 5.8E-13 | 0.218 | 2.43E-31 | 0.047 | 0.013 | 0.034 | 0.069 | 0.010 | 0.605 |
| **rs180349** | - | *BUD13* | 0.23 | 116117037 | 5.8E-30 | 1.25 | 1.7E-07 | 0.050 | 0.008 | 0.133 | 1.2E-12 | 0.207 | 2.23E-28 | 0.043 | 0.022 | 0.021 | 0.264 | 0.014 | 0.441 |
| **rs11066280** | MetS, SBP,TG | *HECTD4* | 0.17 | 111302166 | 5.9E-27 | 0.87 | 2.7E-03 | -0.071 | 5.E-04 | 0.148 | 4.4E-13 | -0.071 | 5.E-04 | -0.100 | 9.85E-07 | -0.098 | 1.59E-06 | -0.076 | 2.E-04 |
| **rs2074356** | HDL | *HECTD4* | 0.15 | 111129784 | 2.2E-25 | 0.86 | 2.8E-03 | -0.081 | 2.E-04 | 0.154 | 1.5E-12 | -0.064 | 0.003 | -0.100 | 3.76E-06 | -0.105 | 1.31E-06 | -0.084 | 1.E-04 |
| **rs17410962** | HDL, TG | *LPL* | 0.12 | 19892360 | 5.8E-23 | 0.79 | 1.4E-05 | -0.033 | 0.161 | -0.197 | 2.0E-17 | -0.172 | 1.27E-13 | -0.039 | 0.091 | -0.014 | 0.554 | 0.021 | 0.370 |
| **rs16940212** | - | *LOC101928635* | 0.34 | 56481312 | 6.1E-23 | 0.91 | 1.7E-02 | 0.019 | 0.256 | -0.136 | 7.3E-17 | 0.038 | 0.020 | -0.006 | 0.722 | -0.018 | 0.272 | 0.005 | 0.743 |
| **rs17482753** | HDL, TG | *LPL* | 0.12 | 19876926 | 1.3E-22 | 0.79 | 1.4E-05 | -0.035 | 0.138 | -0.197 | 2.2E-17 | -0.170 | 2.50E-13 | -0.035 | 0.130 | -0.010 | 0.674 | 0.020 | 0.396 |
| **rs16940174** | HDL | *LOC101928635* | 0.33 | 56473630 | 2.5E-22 | 0.91 | 1.8E-02 | 0.012 | 0.481 | -0.132 | 7.0E-16 | 0.039 | 0.018 | -0.006 | 0.724 | -0.024 | 0.142 | 0.008 | 0.645 |
| **rs10503669** | HDL, TG | *LPL* | 0.12 | 19891970 | 2.8E-22 | 0.79 | 1.9E-05 | -0.034 | 0.149 | -0.197 | 4.0E-17 | -0.171 | 3.09E-13 | -0.037 | 0.117 | -0.013 | 0.588 | 0.020 | 0.387 |
| **rs11216126** | HDL | *BUD13* | 0.20 | 116122450 | 2.6E-21 | 0.82 | 2.1E-05 | 0.006 | 0.771 | -0.160 | 9.4E-17 | -0.162 | 3.79E-17 | 0.012 | 0.529 | 0.003 | 0.896 | -0.013 | 0.503 |
| **rs495348** | HDL | *LOC101928635* | 0.32 | 56475082 | 6.8E-21 | 0.92 | 2.6E-02 | 0.012 | 0.487 | -0.131 | 1.6E-15 | 0.037 | 0.027 | -0.006 | 0.738 | -0.021 | 0.203 | 0.001 | 0.934 |
| **rs16940170** | HDL | *LOC101928635* | 0.33 | 56471574 | 7.3E-21 | 0.91 | 1.6E-02 | 0.012 | 0.468 | -0.132 | 1.2E-15 | 0.034 | 0.042 | -0.009 | 0.582 | -0.024 | 0.140 | 0.005 | 0.778 |
| **rs261301** | HDL | *LOC101928635* | 0.33 | 56474231 | 9.7E-21 | 0.92 | 2.1E-02 | 0.010 | 0.528 | -0.132 | 1.3E-15 | 0.033 | 0.043 | -0.009 | 0.598 | -0.024 | 0.147 | 0.003 | 0.833 |
| **rs12229654** | HDL, MetS | *LOC105369980* | 0.14 | 109898844 | 9.1E-19 | 0.87 | 6.2E-03 | -0.081 | 2.E-04 | 0.134 | 1.1E-09 | -0.055 | 0.013 | -0.080 | 3.E-04 | -0.082 | 2.E-04 | -0.081 | 2.E-04 |
| **rs765547** | - | *LPL* | 0.21 | 19910554 | 9.5E-18 | 0.87 | 1.7E-03 | -0.023 | 0.231 | -0.142 | 6.3E-14 | -0.120 | 2.19E-10 | -0.022 | 0.246 | -0.013 | 0.501 | 0.025 | 0.195 |
| **rs17411126** | HDL, TG | *LPL* | 0.21 | 19899552 | 1.3E-17 | 0.87 | 1.3E-03 | -0.020 | 0.295 | -0.144 | 2.9E-14 | -0.120 | 2.16E-10 | -0.023 | 0.221 | -0.013 | 0.485 | 0.021 | 0.267 |
| **rs4244457** | HDL, TG | *LPL* | 0.33 | 19943326 | 1.7E-17 | 0.85 | 4.2E-05 | -0.015 | 0.372 | -0.135 | 4.3E-16 | -0.116 | 2.63E-12 | -0.006 | 0.718 | -0.007 | 0.670 | -0.007 | 0.658 |
| **rs7013777** | HDL, TG | *LPL* | 0.21 | 19922636 | 2.0E-17 | 0.86 | 6.8E-04 | -0.024 | 0.205 | -0.141 | 9.4E-14 | -0.121 | 1.86E-10 | -0.024 | 0.207 | -0.021 | 0.259 | 0.022 | 0.245 |
| **rs11986942** | HDL, TG | *LPL* | 0.21 | 19911725 | 2.3E-17 | 0.87 | 1.7E-03 | -0.022 | 0.242 | -0.141 | 8.0E-14 | -0.119 | 2.73E-10 | -0.024 | 0.212 | -0.014 | 0.449 | 0.022 | 0.238 |
| **rs1919484** | HDL, TG | *LPL* | 0.21 | 19913956 | 2.3E-17 | 0.88 | 2.4E-03 | -0.021 | 0.273 | -0.141 | 9.5E-14 | -0.119 | 3.45E-10 | -0.022 | 0.238 | -0.013 | 0.489 | 0.024 | 0.199 |
| **rs17411031** | HDL, TG | *LPL* | 0.21 | 19896590 | 2.4E-17 | 0.87 | 1.7E-03 | -0.018 | 0.334 | -0.144 | 3.1E-14 | -0.119 | 3.76E-10 | -0.026 | 0.174 | -0.016 | 0.406 | 0.020 | 0.290 |
| **rs1837842** | HDL, TG | *LPL* | 0.21 | 19912570 | 2.6E-17 | 0.87 | 1.4E-03 | -0.021 | 0.260 | -0.141 | 8.0E-14 | -0.120 | 2.64E-10 | -0.022 | 0.243 | -0.013 | 0.481 | 0.022 | 0.238 |
| **rs4922117** | HDL, TG | *LPL* | 0.21 | 19896866 | 3.7E-17 | 0.87 | 1.4E-03 | -0.020 | 0.292 | -0.143 | 3.9E-14 | -0.117 | 5.91E-10 | -0.025 | 0.185 | -0.015 | 0.426 | 0.020 | 0.285 |
| **rs6586891** | HDL, TG | *LPL* | 0.32 | 19958878 | 4.7E-17 | 0.87 | 2.0E-04 | -0.011 | 0.516 | -0.132 | 1.8E-15 | -0.113 | 1.07E-11 | -0.006 | 0.704 | -0.008 | 0.647 | -5.E-04 | 0.978 |
| **rs7461115** | HDL, TG | *LPL* | 0.21 | 19915820 | 8.0E-17 | 0.86 | 8.4E-04 | -0.024 | 0.211 | -0.140 | 2.1E-13 | -0.120 | 3.41E-10 | -0.020 | 0.304 | -0.016 | 0.393 | 0.022 | 0.242 |
| **rs17489282** | HDL, TG | *LPL* | 0.21 | 19896798 | 1.4E-16 | 0.86 | 7.2E-04 | -0.022 | 0.254 | -0.139 | 1.4E-13 | -0.119 | 2.77E-10 | -0.031 | 0.096 | -0.019 | 0.320 | 0.014 | 0.457 |
| **rs603446** | TG | *ZPR1* | 0.23 | 116159645 | 4.4E-16 | 0.91 | 3.6E-02 | -0.010 | 0.574 | -0.094 | 3.4E-07 | -0.154 | 5.86E-17 | -0.020 | 0.288 | -0.030 | 0.108 | 7.E-04 | 0.969 |
| **rs271** | HDL, TG | *LPL* | 0.21 | 19857982 | 5.7E-12 | 0.89 | 1.1E-02 | 0.007 | 0.708 | -0.115 | 1.1E-09 | -0.090 | 1.85E-06 | -0.015 | 0.429 | -0.005 | 0.801 | 0.037 | 0.049 |
| **rs4420638** | HDL, LDL,TG | *APOC1* | 0.11 | 50114786 | 6.6E-12 | 1.23 | 1.5E-04 | 0.039 | 0.107 | 0.161 | 3.6E-11 | 0.141 | 6.31E-09 | -0.027 | 0.276 | -0.011 | 0.645 | 0.020 | 0.419 |
| **rs263** | HDL,TG | *LPL* | 0.21 | 19857092 | 1.9E-11 | 0.90 | 1.7E-02 | 0.007 | 0.711 | -0.112 | 3.4E-09 | -0.087 | 3.95E-06 | -0.017 | 0.358 | -0.007 | 0.710 | 0.038 | 0.044 |
| **rs780092** | TG | *GCKR* | 0.33 | 27596658 | 3.3E-11 | 0.89 | 3.4E-03 | 0.013 | 0.431 | 0.009 | 5.8E-01 | -0.108 | 6.46E-11 | -0.017 | 0.292 | -0.005 | 0.765 | -0.036 | 0.032 |
| **rs12279433** | - | *SIK3* | 0.10 | 116253431 | 1.4E-10 | 0.88 | 3.9E-02 | -0.001 | 0.970 | -0.086 | 9.0E-04 | -0.179 | 4.05E-12 | -0.042 | 0.103 | -0.026 | 0.320 | 0.010 | 0.707 |
| **rs17120119** | - | *SIK3* | 0.11 | 116252492 | 2.3E-10 | 0.89 | 4.7E-02 | 0.030 | 0.239 | -0.083 | 1.2E-03 | -0.164 | 9.51E-11 | -0.041 | 0.108 | -0.026 | 0.307 | 0.024 | 0.350 |
| **rs2044426** | - | *SIK3* | 0.10 | 116261393 | 2.7E-10 | 0.88 | 3.7E-02 | 0.001 | 0.979 | -0.084 | 1.1E-03 | -0.178 | 6.01E-12 | -0.040 | 0.119 | -0.025 | 0.337 | 0.008 | 0.758 |
| **rs6589567** | LDL,TG | *APOA5* | 0.27 | 116175886 | 1.1E-09 | 1.10 | 1.8E-02 | 0.031 | 0.075 | 0.080 | 4.4E-06 | 0.112 | 9.05E-11 | 0.011 | 0.534 | 0.023 | 0.187 | 0.013 | 0.446 |
| **rs6494005** | HDL | *LOC101928635* | 0.27 | 56511816 | 3.1E-09 | 0.92 | 2.9E-02 | 0.010 | 0.572 | 0.092 | 1.3E-07 | -0.033 | 0.054 | -0.023 | 0.190 | -0.028 | 0.108 | 0.007 | 0.684 |
| **rs3782889** | coronary heart disease | *MYL2* | 0.17 | 109835038 | 4.2E-09 | 0.87 | 3.7E-03 | -0.071 | 5.E-04 | 0.071 | 4.7E-04 | -0.033 | 0.109 | -0.060 | 0.003 | -0.050 | 0.014 | -0.076 | 2.E-04 |
| **rs486394** | TG | *LOC101929011* | 0.12 | 116031532 | 5.8E-09 | 1.19 | 1.1E-03 | 0.032 | 0.178 | 0.097 | 3.5E-05 | 0.128 | 5.07E-08 | 0.064 | 0.006 | 0.051 | 0.029 | -0.014 | 0.556 |
| **rs11065756** | T2D | *CCDC63* | 0.17 | 109823177 | 9.6E-09 | 0.88 | 8.9E-03 | -0.067 | 0.001 | 0.072 | 3.9E-04 | -0.032 | 0.116 | -0.059 | 0.004 | -0.048 | 0.019 | -0.073 | 4.E-04 |
| **rs10849915** | alcohol | *CCDC63* | 0.18 | 109818005 | 1.8E-08 | 0.88 | 7.9E-03 | -0.064 | 0.002 | 0.068 | 7.6E-04 | -0.031 | 0.127 | -0.060 | 0.003 | -0.044 | 0.029 | -0.074 | 2.E-04 |
| **rs4351435** | - | *LOC105375746* | 0.30 | 126569060 | 2.2E-08 | 1.08 | 3.9E-02 | 0.011 | 0.509 | -3.E-04 | 9.9E-01 | 0.099 | 6.02E-09 | 0.023 | 0.175 | 0.028 | 0.103 | -0.011 | 0.525 |
| **rs1800775** | HDL,LDL,TG | *CETP* | 0.46 | 55552737 | 2.8E-08 | 1.09 | 1.5E-02 | -8.24E-05 | 0.996 | 0.099 | 1.4E-10 | 0.034 | 0.030 | 0.010 | 0.512 | 0.019 | 0.227 | 0.003 | 0.846 |
| **rs7396851** | TG | *APOA4* | 0.32 | 116189374 | 3.4E-08 | 1.09 | 2.9E-02 | 0.020 | 0.236 | 0.036 | 3.4E-02 | 0.101 | 1.55E-09 | -0.012 | 0.466 | -0.029 | 0.089 | 0.017 | 0.301 |
| **rs7396835** | TG | *APOA4* | 0.32 | 116189238 | 3.6E-08 | 1.09 | 2.9E-02 | 0.020 | 0.234 | 0.036 | 3.1E-02 | 0.101 | 1.58E-09 | -0.012 | 0.477 | -0.028 | 0.090 | 0.017 | 0.301 |
| **rs12708980** | HDL | *CETP* | 0.10 | 55569880 | 6.0E-08 | 1.12 | 4.7E-02 | 0.038 | 0.138 | 0.162 | 3.2E-10 | 0.046 | 0.074 | -0.007 | 0.773 | -0.015 | 0.555 | 0.027 | 0.290 |
| **rs17482310** | HDL, TG | *LOC105379309* | 0.12 | 19791156 | 1.5E-07 | 0.84 | 1.3E-03 | 0.008 | 0.724 | -0.102 | 1.3E-05 | -0.124 | 1.08E-07 | -0.034 | 0.143 | -0.012 | 0.604 | 0.004 | 0.855 |

Chr, chromosome; CA, coded allele; NCA, noncoded allele; MulA_P, multivariate analysis p value; OR, odds ratio; LR, logistic analysis;FAG, fast glucose level; TG, triglyceride; WC, waist circumference

Supplementary Table S2. The newly identified common variants for metabolic syndrome in the replication stage.

| **SNP** | **Traits** | **Nearest Gene** | **Chr** | **MAF** | **Position** | **MulA_p** | **OR** | **OR_p** | **FAG** | | **-HDLc** | | **TG** | | **SBP** | | **DBP** | | **WC** | |
| --- | --- | --- | --- | --- | --- | --- | --- | --- | --- | --- | --- | --- | --- | --- | --- | --- | --- | --- | --- | --- |
|  |  |  |  |  |  |  |  |  | **beta** | **p** | **beta** | **p** | **beta** | **p** | **beta** | **p** | **beta** | **p** | **beta** | **p** |
| **rs6589566** | TG | *ZPR1* | 11 | 0.216 | 116157633 | 2.1E-14 | 1.188 | 0.036 | -0.045 | 0.112 | 0.145 | 2.6E-07 | 0.195 | 3.7E-12 | -0.023 | 0.415 | -0.009 | 0.752 | -0.026 | 0.349 |
| **rs17410962** | HDL, TG | *LPL* | 8 | 0.119 | 19892360 | 6.6E-12 | 0.681 | 0.002 | -0.014 | 0.690 | -0.276 | 1.7E-14 | -0.164 | 5.4E-06 | -0.016 | 0.655 | 0.012 | 0.731 | -0.067 | 0.063 |
| **rs17482753** | HDL, TG | *LPL* | 8 | 0.119 | 19876926 | 7.5E-12 | 0.663 | 0.001 | -0.019 | 0.604 | -0.277 | 1.7E-14 | -0.170 | 2.5E-06 | -0.024 | 0.508 | -0.001 | 0.980 | -0.068 | 0.062 |
| **rs10503669** | HDL, TG | *LPL* | 8 | 0.118 | 19891970 | 1.2E-11 | 0.685 | 0.002 | -0.016 | 0.659 | -0.274 | 2.6E-14 | -0.163 | 6.4E-06 | -0.017 | 0.631 | 0.009 | 0.794 | -0.067 | 0.065 |
| **rs11216126** | HDL | *BUD13* | 11 | 0.193 | 116122450 | 7.3E-09 | 0.772 | 0.006 | 0.067 | 0.024 | -0.118 | 6.4E-05 | -0.129 | 1.1E-05 | 0.070 | 0.017 | 0.047 | 0.110 | 0.039 | 0.190 |
| **rs765547** | - | *LPL* | 8 | 0.201 | 19910554 | 3.9E-07 | 0.737 | 0.001 | -0.017 | 0.559 | -0.165 | 1.3E-08 | -0.129 | 8.9E-06 | 0.012 | 0.679 | 0.026 | 0.373 | -0.042 | 0.150 |
| **rs17411126** | HDL, TG | *LPL* | 8 | 0.199 | 19899552 | 8.0E-08 | 0.705 | 0.000 | -0.018 | 0.532 | -0.171 | 4.3E-09 | -0.140 | 1.4E-06 | 0.007 | 0.813 | 0.022 | 0.458 | -0.047 | 0.106 |
| **rs4244457** | HDL, TG | *LPL* | 8 | 0.326 | 19943326 | 5.5E-06 | 0.729 | 0.000 | -0.039 | 0.117 | -0.127 | 3.8E-07 | -0.110 | 1.0E-05 | -0.006 | 0.796 | -0.008 | 0.741 | -0.025 | 0.323 |
| **rs7013777** | HDL, TG | *LPL* | 8 | 0.201 | 19922636 | 1.4E-07 | 0.742 | 0.002 | -0.013 | 0.657 | -0.172 | 3.5E-09 | -0.131 | 6.7E-06 | 0.004 | 0.902 | 0.018 | 0.545 | -0.036 | 0.215 |
| **rs11986942** | HDL, TG | *LPL* | 8 | 0.202 | 19911725 | 2.0E-07 | 0.731 | 0.001 | -0.016 | 0.582 | -0.166 | 8.9E-09 | -0.129 | 8.2E-06 | 0.015 | 0.605 | 0.031 | 0.285 | -0.041 | 0.153 |
| **rs1919484** | HDL, TG | *LPL* | 8 | 0.201 | 19913956 | 3.7E-07 | 0.734 | 0.001 | -0.019 | 0.521 | -0.165 | 1.4E-08 | -0.129 | 8.0E-06 | 0.012 | 0.675 | 0.025 | 0.381 | -0.041 | 0.157 |
| **rs17411031** | HDL, TG | *LPL* | 8 | 0.201 | 19896590 | 2.2E-07 | 0.717 | 0.000 | -0.016 | 0.587 | -0.164 | 1.6E-08 | -0.133 | 4.4E-06 | 0.014 | 0.628 | 0.031 | 0.283 | -0.042 | 0.146 |
| **rs1837842** | HDL, TG | *LPL* | 8 | 0.202 | 19912570 | 3.3E-07 | 0.730 | 0.001 | -0.017 | 0.550 | -0.165 | 1.3E-08 | -0.131 | 6.7E-06 | 0.012 | 0.677 | 0.026 | 0.373 | -0.043 | 0.135 |
| **rs4922117** | HDL, TG | *LPL* | 8 | 0.201 | 19896866 | 1.7E-07 | 0.721 | 0.001 | -0.016 | 0.580 | -0.165 | 1.2E-08 | -0.133 | 4.6E-06 | 0.012 | 0.673 | 0.031 | 0.281 | -0.041 | 0.159 |
| **rs6586891** | HDL, TG | *LPL* | 8 | 0.322 | 19958878 | 6.7E-06 | 0.723 | 0.000 | -0.041 | 0.103 | -0.124 | 7.5E-07 | -0.114 | 5.1E-06 | -0.003 | 0.892 | -0.002 | 0.931 | -0.030 | 0.223 |
| **rs7461115** | HDL, TG | *LPL* | 8 | 0.199 | 19915820 | 2.5E-07 | 0.755 | 0.003 | -0.015 | 0.599 | -0.169 | 5.7E-09 | -0.125 | 1.8E-05 | 0.014 | 0.622 | 0.026 | 0.380 | -0.034 | 0.249 |
| **rs17489282** | HDL, TG | *LPL* | 8 | 0.200 | 19896798 | 3.0E-07 | 0.722 | 0.001 | -0.017 | 0.552 | -0.164 | 1.6E-08 | -0.130 | 7.1E-06 | 0.013 | 0.649 | 0.030 | 0.296 | -0.043 | 0.139 |
| **rs271** | HDL, TG | *LPL* | 8 | 0.201 | 19857982 | 7.9E-05 | 0.723 | 0.001 | 0.005 | 0.864 | -0.141 | 1.5E-06 | -0.092 | 0.002 | 0.021 | 0.485 | 0.037 | 0.204 | -0.034 | 0.254 |
| **rs263** | HDL,TG | *LPL* | 8 | 0.202 | 19857092 | 9.5E-05 | 0.716 | 0.000 | 0.005 | 0.867 | -0.141 | 1.5E-06 | -0.089 | 0.002 | 0.024 | 0.419 | 0.039 | 0.190 | -0.040 | 0.177 |
| **rs7396851** | TG | *APOA4* | 11 | 0.331 | 116189374 | 0.004 | 1.163 | 0.038 | -0.021 | 0.391 | 0.018 | 0.4651 | 0.080 | 0.001 | 0.015 | 0.547 | -0.003 | 0.891 | -0.033 | 0.178 |
| **rs7396835** | TG | *APOA4* | 11 | 0.331 | 116189238 | 0.004 | 1.164 | 0.037 | -0.021 | 0.398 | 0.018 | 0.4719 | 0.080 | 0.001 | 0.015 | 0.540 | -0.003 | 0.892 | -0.033 | 0.178 |
| **rs765547** | - | *LPL* | 8 | 0.201 | 19910554 | 3.9E-07 | 0.737 | 0.001 | -0.017 | 0.559 | -0.165 | 1.3E-08 | -0.129 | 0.9E-05 | 0.012 | 0.679 | 0.026 | 0.373 | -0.042 | 0.150 |
| **rs6589567** | LDL,TG | *APOA5* | 11 | 0.275 | 116175886 | 2.3E-05 | 0.984 | 0.843 | -0.043 | 0.101 | 0.061 | 0.020 | 0.099 | 0.1E-03 | -0.012 | 0.658 | -0.018 | 0.479 | -0.048 | 0.063 |
| **rs6494005** | HDL | *LIPC* | 15 | 0.257 | 56511816 | 0.001 | 1.055 | 0.504 | -0.042 | 0.115 | 0.070 | 0.008 | -0.054 | 0.043 | 0.006 | 0.816 | 0.006 | 0.937 | -0.006 | 0.815 |
| **rs3782889** | coronary heart disease | *MYL2* | 12 | 0.172 | 109835038 | 0.014 | 0.957 | 0.640 | -0.037 | 0.233 | 0.106 | 0.6E-03 | 0.045 | 0.144 | -0.015 | 0.635 | 0.012 | 0.699 | 0.008 | 0.801 |
| **rs11065756** | T2D | *CCDC63* | 12 | 0.172 | 109823177 | 0.011 | 0.967 | 0.720 | -0.041 | 0.192 | 0.107 | 0.5E-03 | 0.047 | 0.133 | -0.018 | 0.055 | 0.010 | 0.754 | 0.009 | 0.766 |
| **rs10849915** | alcohol | *CCDC63* | 12 | 0.172 | 109818005 | 0.019 | 0.963 | 0.691 | -0.042 | 0.178 | 0.101 | 0.1E-02 | 0.036 | 0.249 | -0.024 | 0.434 | 0.002 | 0.950 | 0.002 | 0.938 |
| **rs12708980** | HDL | *CETP* | 16 | 0.099 | 55569880 | 0.4E-03 | 0.972 | 0.810 | -0.032 | 0.404 | 0.145 | 0.1E-03 | -0.016 | 0.681 | -0.045 | 0.240 | -0.004 | 0.909 | -0.031 | 0.427 |

Chr, chromosome; CA, coded allele; NCA, noncoded allele; MulA_P, multivariate analysis p value; OR, odds ratio; FAG, fast glucose level; TG, triglyceride; WC, waist circumference

Supplementary Table S3. The newly identified common variants for metabolic syndrome in the combined meta-analysis.

| **SNP** | **Traits** | **Nearest Gene** | **Chr** | **CA/NCA** | **MAF** | **Position** | **MulA_P** | **OR** | **OR_P** | **FAG** | | **-HDLc** | | **TG** | | **SBP** | | **DBP** | | **WC** | |
| --- | --- | --- | --- | --- | --- | --- | --- | --- | --- | --- | --- | --- | --- | --- | --- | --- | --- | --- | --- | --- | --- |
|  |  |  |  |  |  |  |  |  |  | **beta** | **p** | **beta** | **p** | **beta** | **p** | **beta** | **p** | **beta** | **p** | **beta** | **p** |
| rs16940212 | HDL | *LOC101928635* | 15 | T/G | 0.34 | 56481312 | 3.80E-20 | 0.92 | 0.016 | 0.02 | 0.08 | -0.11 | 1.6E-16 | 0.02 | 0.092 | -0.003 | 0.84 | -0.01 | 0.53 | 0.00 | 0.87 |
| rs495348 | HDL | *LOC101928635* | 15 | G/C | 0.33 | 56475082 | 2.59E-18 | 0.93 | 0.031 | 0.02 | 0.12 | -0.11 | 9.3E-16 | 0.02 | 0.162 | -0.001 | 0.96 | -0.01 | 0.45 | 0.00 | 0.83 |
| rs16940170 | HDL | *LOC101928635* | 15 | A/G | 0.33 | 56471574 | 4.49E-18 | 0.92 | 0.016 | 0.02 | 0.15 | -0.11 | 1.1E-15 | 0.02 | 0.216 | -0.004 | 0.76 | -0.01 | 0.32 | 0.00 | 0.95 |
| rs780094 | MetS, TG,HDL,LDL | *GCKR* | 2 | C/T | 0.46 | 27594741 | 3.15E-09 | 0.94 | 0.042 | 0.03 | 0.02 | 0.00 | 0.9 | -0.07 | 3.7E-08 | 0.002 | 0.90 | 0.01 | 0.35 | 0.01 | 0.63 |
| rs486394 | TG | *LOC101929011* | 11 | C/A | 0.12 | 116031532 | 7.53E-08 | 1.16 | 0.002 | 0.02 | 0.25 | 0.08 | 7.2E-05 | 0.10 | 1.0E-06 | 0.054 | 0.01 | 0.04 | 0.05 | -0.01 | 0.47 |
| rs17482310 | HDL,TG | *LOC105379309* | 8 | T/G | 0.12 | 19791156 | 7.94E-08 | 0.89 | 0.017 | -0.01 | 0.46 | -0.09 | 9.0E-06 | -0.11 | 8.0E-08 | -0.037 | 0.06 | -0.02 | 0.29 | 0.00 | 0.89 |

Chr, chromosome; CA, coded allele; NCA, noncoded allele; MulA_P, multivariate analysis p value; OR, odds ratio; FAG, fast glucose level; TG, triglyceride; WC, waist circumference

Supplementary Table S4. Association of rare variants with metabolic syndrome using gene-based test in the discovery, replication and combined meta-analysis

| **Gene** | **Chr** | **Position (hg19)** | **KARE** | **HEXA** | **Meta-Analysis** |
| --- | --- | --- | --- | --- | --- |
| *CETP* | 16 | 57007387 | 1.39E-32 | 1.33E-16 | 2.05E-46 |
| *SH2B1* | 16 | 28878202 | 1.47E-06 | 1.000 | 2.12E-05 |
| *ZFP2* | 5 | 178359696 | 3.08E-06 | 1.000 | 4.22E-05 |
| *MTRR* | 5 | 7870973 | 7.48E-06 | 1.000 | 9.57E-05 |
| *ATOH1* | 4 | 94750491 | 2.E-04 | 1.000 | 0.002 |
| *ABCA6* | 17 | 67077237 | 3.E-04 | 0.902 | 0.002 |
| *SGK223* | 8 | 8185290 | 3.E-04 | 0.242 | 0.001 |
| *HERC4* | 10 | 69750901 | 4.E-04 | 0.486 | 0.002 |
| *TOP1MT* | 8 | 144398191 | 5.E-04 | 0.481 | 0.002 |
| *HLA-DRB1* | 6 | 32552121 | 0.001 | 0.128 | 0.001 |
| *FAM118A* | 22 | 45723846 | 0.001 | 1.000 | 0.005 |
| *PRPF6* | 20 | 62663363 | 0.001 | 1.000 | 0.005 |
| *TOB2* | 22 | 41832844 | 0.001 | 1.000 | 0.005 |
| *AHCTF1* | 1 | 247006051 | 0.001 | 0.073 | 5.E-04 |
| *TYRP1* | 9 | 12694066 | 0.001 | 0.913 | 0.005 |
| *CGRRF1* | 14 | 54996872 | 0.001 | 1.000 | 0.006 |
| *G6PC2* | 2 | 169761042 | 0.001 | 0.735 | 0.005 |
| *DLK1* | 14 | 101198426 | 0.001 | 0.057 | 5.E-04 |
| *CRISP1* | 6 | 49806156 | 0.001 | 0.639 | 0.004 |
| *PEG10* | 7 | 94294682 | 0.001 | 0.570 | 0.004 |

Chr, chromosome

Supplementary Table S5. Results of association of the observed *CETP* rare variants with metabolic syndrome and its traits

| Gene | SNP | Amino acid change | rs number | Chr | location | CA/NCA | MAF | FAG | | -HDLc | | TG | | SBP | | DBP | | **WC** | |
| --- | --- | --- | --- | --- | --- | --- | --- | --- | --- | --- | --- | --- | --- | --- | --- | --- | --- | --- | --- |
|  |  |  |  |  |  |  |  | beta | p | beta | p | beta | p | beta | p | beta | p | beta | p |
| **KARE** | | |  |  |  |  |  |  |  |  |  |  |  |  |  |  |  |  |  |
| *CETP* | exm1243004 | exon15,A1376G,D459G | rs2303790 | 16 | 57017292 | G/A | 0.046 | -0.04 | 0.33 | -0.45 | 1.47E-28 | -0.02 | 0.58 | -0.03 | 0.53 | 0.02 | 0.69 | 0.01 | 0.87 |
| *CETP* | exm1242973 | exon11,G991A,G331S | rs5881 | 16 | 57012012 | A/G | 0.003 | -0.20 | 0.24 | 0.32 | 0.06 | -0.04 | 0.82 | -0.28 | 0.09 | -0.24 | 0.15 | -0.09 | 0.59 |
| *CETP* | exm1242986 | exon12, G1168C,A390P | rs5880 | 16 | 57015091 | C/G | 4.E-04 | 0.29 | 0.47 | 0.31 | 0.45 | -0.05 | 0.89 | -0.16 | 0.68 | -0.27 | 0.50 | 0.18 | 0.67 |
| *CETP* | exm1242967 | exon10, G940A,E314K | rs140547417 | 16 | 57007387 | A/G | 7.47E-05 | 0.37 | 0.71 | 0.47 | 0.64 | 0.91 | 0.36 | 0.29 | 0.77 | 0.32 | 0.75 | 0.40 | 0.69 |
| **HEXA** | | |  |  |  |  |  |  |  |  |  |  |  |  |  |  |  |  |  |
| *CETP* | exm1243004 | exon15,A1376G,D459G | rs2303790 | 16 | 57017292 | G/A | 0.046 | -0.09 | 0.15 | -0.45 | 6.05E-15 | -0.04 | 0.47 | -0.03 | 0.65 | 0.03 | 0.66 | 0.04 | 0.51 |
| *CETP* | exm1242973 | exon11,G991A,G331S | rs5881 | 16 | 57012012 | A/G | 0.003 | 0.32 | 0.15 | 0.07 | 0.75 | -0.03 | 0.89 | -0.37 | 0.09 | -0.20 | 0.37 | -0.03 | 0.90 |
| *CETP* | exm1242986 | exon12, G1168C,A390P | rs5880 | 16 | 57015091 | C/G | 0.001 | 0.30 | 0.38 | 0.84 | 0.01 | 0.10 | 0.76 | -0.20 | 0.55 | -0.06 | 0.87 | -0.29 | 0.38 |
| **Meta Analysis** | | |  |  |  |  |  |  |  |  |  |  |  |  |  |  |  |  |  |
| *CETP* | exm1243004 | exon15,A1376G,D459G | rs2303790 | 16 | 57017292 | G/A | 0.046 | -0.06 | 0.19 | -0.45 | 8.69E-41 | -0.03 | 0.63 | -0.03 | 0.71 | 0.02 | 0.81 | 0.02 | 0.80 |
| *CETP* | exm1242973 | exon11,G991A,G331S | rs5881 | 16 | 57015091 | A/G | 0.003 | 0.06 | 0.15 | 0.20 | 0.18 | -0.03 | 0.96 | -0.33 | 0.05 | -0.22 | 0.21 | -0.06 | 0.87 |
| *CETP* | exm1242986 | exon12, G1168C,A390P | rs5880 | 16 | 57012012 | C/G | 7.41.E-04 | 0.29 | 0.48 | 0.57 | 0.03 | 0.02 | 0.94 | -0.18 | 0.74 | -0.16 | 0.80 | -0.06 | 0.60 |

Chr, chromosome; CA, coded allele; NCA, noncoded allele; MulA_P, multivariate analysis p value; OR, odds ratio; FAG, fast glucose level; TG, triglyceride; WC, waist circumference

Supplementary Table S6. SNP and Exom Chip genotyping, data cleaning and quality control

| Study | Genotype Array | Sample QC Exclusion Criteria | SNP QC Exclusion Criteria | Statistical Analysis Methods | Final Sample Size for Statistical Analysis |
| --- | --- | --- | --- | --- | --- |
| KARE | AFFY 5.0 | more than one missing phenotype | SNP call rate <95%; HWE p-value < 10E-6 | Multivariate regression: MANOVA with wilks statistics by R; single variant analysis: plink ver 1.0.3. linear option ; logistic regression: plink ver 1.0.3. logistic option | 8,373 (case: 1,946 , control: 6,427) |
|  | Exome chip array | more than one missing phenotype | SNP call rate <95%; MAF <5% ; MAC>2 | Multivariate analysis: MAAUSS by R ; single variant analysis: SKAT package in R | 6,693 (case: 1,530 , control: 5,163) |
| HEXA | AFFY 6.0 | more than one missing phenotype | SNP call rate <95%; HWE p-value < 10E-6 | Multivariate regression: MANOVA with wilks statistics by R; single variant analysis: plink ver 1.0.3. linear option ; logistic regression: plink ver 1.0.3. logistic option | 3,694 (case: 430, control: 3,264) |
|  | Exome chip array | more than one missing phenotype | SNP call rate <95%; MAF <5% ; MAC>2 | Multivariate analysis: MAAUSS by R ; single variant analysis: SKAT package in R | 3,429 (case: 411, control:3,108) |

AFFY 5.0, Affymetrics genotyping array-Genome-Wide Human SNP Array 5.0; AFFY 6.0, Affymetrics genotyping array-Genome-Wide Human SNP Array 6.0; MAF, minor allele frequency; MAC, minor allele count; MANOVA, multivariate analysis of variace; MAAUSS, Multivariate Association Analysis using Score Statistics
